# Machine intelligence today: applications, methodology, and technology: Selected results of the 1st online Dagstuhl workshop on applied machine intelligence

**DOI:** 10.1007/s00287-021-01343-1

**Published:** 2021-02-18

**Authors:** Bernhard G. Humm, Hermann Bense, Michael Fuchs, Benjamin Gernhardt, Matthias Hemmje, Thomas Hoppe, Lukas Kaupp, Sebastian Lothary, Kai-Uwe Schäfer, Bernhard Thull, Tobias Vogel, Rigo Wenning

**Affiliations:** 1 Hochschule Darmstadt—University of Applied Sciences, Haardtring 100, 64295 Darmstadt, Germany; 2 bense.com GmbH, Schwarze-Brüder-Str. 1, 44137 Dortmund, Germany; 3 Wilhelm Büchner Hochschule, Hilpertstraße 31, 64295 Darmstadt, Germany; 4 FernUniversität Hagen, Universitätsstr. 1, 58097 Hagen, Germany; 5 Fraunhofer FOKUS, Kaiserin-Augusta-Allee 31, 10589 Berlin, Germany; 6 Hochschule für Technik und Wirtschaft Berlin, 12459 Wilhelminenhofstr. 75a, Germany; 7 Frankfurt am Main, Germany; 8 World Wide Web Consortium, 2004 Route des Lucioles, 06902 Sophia Antipolis, France

## Abstract

Machine intelligence, a.k.a. artificial intelligence (AI) is one of the most prominent and relevant technologies today. It is in everyday use in the form of AI applications and has a strong impact on society. This article presents selected results of the 2020 Dagstuhl workshop on applied machine intelligence. Selected AI applications in various domains, namely culture, education, and industrial manufacturing are presented. Current trends, best practices, and recommendations regarding AI methodology and technology are explained. The focus is on ontologies (knowledge-based AI) and machine learning.

## Introduction

*Machine intelligence*, a.k.a. *artificial intelligence (AI)*^1^, is one of the most prominent and relevant technologies today. It is in everyday use in the form of AI applications and has a strong impact on society.

In 2014, we started a series of annual workshops at the Leibniz Zentrum für Informatik, Schloss Dagstuhl, Germany, initially focusing on corporate semantic web, and later widening the scope to applied machine intelligence (AMI). In all workshops, we focussed on the application of AI technologies in corporate and organizational contexts. A number of books [1–3] and journal articles [4–7] resulted from those workshops. The workshops are characterized by an intense spirit of interdisciplinarity, collaboration, and focus on practical results [8]. Due to the coronavirus pandemic, the 2020 workshop was, for the first time, held online—however, this made it no less intense. It consisted of two half-day workshops with short presentations and parallel barcamp sessions. This article presents selected results from the 2020 workshop.

This article is structured as follows: In the next section we present selected AI applications in various domains, namely culture, education, and industrial manufacturing. The following section focuses on AI methodology, namely aspects of machine learning and knowledge representation. We then discuss selected technological issues of AI before concluding this article.

## Current applications of AI

The following overview of AI applications in the domains of culture, education, and industrial manufacturing is not meant to be exhaustive, but shall demonstrate the diversity of AI by examples.

### AI for performing arts

**Fig. 1:**
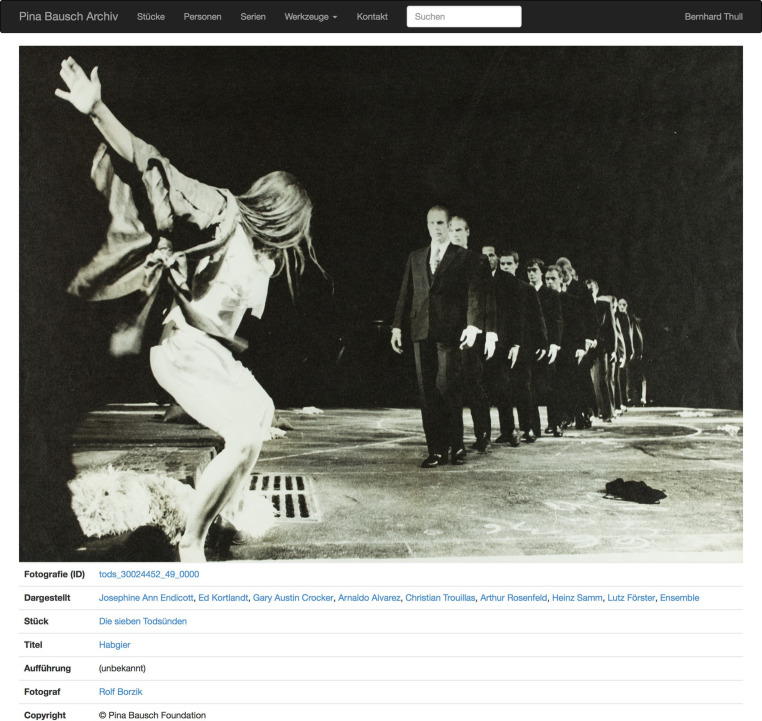
Data browser for the digital archive of the Pina Bausch Foundation with a web page containing data on a photograph. By clicking on the corresponding links, users can find out more about the photograph itself, people depicted therein, the piece “The Seven Deadly Sins of the Petty Bourgeoisie,” the title or the scene “Greed,” and the photographer Rolf Borzik

The term *performing arts* refers to ephemeral forms of art in which artists use their voices, bodies, or inanimate objects to convey artistic expression^2^. This comprises opera, theatre, ballet, concerts, and many other types of performances. As part of our cultural heritage, it is important to preserve works of performing arts. Developing archives for performing arts involves particular challenges. Due to its ephemeral nature, it is not possible to archive the performance itself. Instead, archives of performing arts contain artefacts in which a performing art work manifests itself, e.g., photographs, videos of performances, newspaper reviews, or interviews with contemporary witnesses (oral history). For major works, this leads to a large amount of typically unstructured material, which must be made accessible by archivists and historians. Examples of projects that have dealt with the development of archives for performing arts include:

- Development of the digital Pina Bausch archive of the Pina Bausch Foundation, Wuppertal, Germany (2011–2020): Development of an archive to represent the work of the choreographer Pina Bausch based on standards of Linked Data and Semantic Web, as well as CIDOC/Conceptual Reference Model (CRM) [9]. See Fig. 1.
- Development of the archive of the free theatre (dt. Archiv des Freien Theaters, 2017): Development of an approach to archive the work of more than 3000 individual artists and small companies who have performed theatre in Germany since the 1970s. The approach was also based on semantic web standards [10].
- Project study on the implementation of a live archive for the David Earle Dance Theatre, Toronto, Canada (2018–2019): Use of an archive to create new performances based on archived data by mixing real performances with archived material.

Which AI lessons can be learned from the development of archives for performing arts? Semantic web standards have proven to be well suited for these tasks. Additionally, we identified the need to apply pattern recognition algorithms. Due to high demands on data quality and accuracy, retrieval and formalization of data is done manually. However, this cannot usually be done for all collected material, the archive of the free theatre being the most striking example. It is therefore necessary to identify promising candidates within a huge search space, i.e, material that has not yet been described, but whose cataloguing is likely worthwhile. Here, the application of automated pattern recognition is a promising approach.

In the Pina Bausch Archive, videos are analyzed by marking scenes within a recorded performance. With more than 8000 video tapes and sometimes more than 100 scenes within one performance, this leads to a huge amount of work when carried out manually. In performances, scenes are usually identified with the help of cues, i.e., certain movements of actors, or changes in lighting or music. Automatic cue detection on videos can be of help here.

The reuse of material, e.g., to create new performances, creates the need to synchronize activities in real time. This is likewise done with the help of cues, and again, automatic cue detection on video and onstage is helpful.

Archiving material is a task lasting many years, carried out by institutions that are typically not equipped with IT departments. It is therefore a mandatory prerequisite that machine intelligence applications can be configured and used by lay people.

### Building a semantic qualification web

The opportunities and offers for qualification and life-long learning are becoming ever more extensive. If you consider the academic sector alone, there are already more than 20,000 study courses offered by approximately 400 qualification institutes in Germany [11]. Consequently, planning individual qualifications may become a challenge. How might AI help in this scenario?

The vision of Tim Berners-Lee^3^, the inventor of the WWW, was to create a data network in which software agents (SWA) can act [12]. SWAs are symbolic AI programs that can navigate the WWW and make autonomous decisions based on ontologies [13]. Examples of SWA tasks include booking trips, arranging medical appointments, or creating qualification paths, i.e., sequences of qualifying actions that lead to a certain qualification goal. This data network was named *semantic web (SeW)* and was introduced by Berners-Lee in the Scientific American 2002 [14, 15].

We use the term *semantic qualification web (SQW)* for the idea of a semantic network in the qualification sector. An SQW needs to model qualifications offered by organizations (e.g., universities, courses, and modules), as well as competences taught. Such information is usually provided in textual form on the websites of such organizations, e.g., in module descriptions. A representation in machine-readable form, e.g, as an ontology using SeW standards, is rare. Manually creating such an SQW ontology is extremely cost-intensive and constantly keeping it up-to-date is not feasible. Therefore, AI methods of natural language processing (NLP) using machine learning (ML) may be used to semi-automatically create an SQW ontology from texts provided on such websites.

**Fig. 2:**
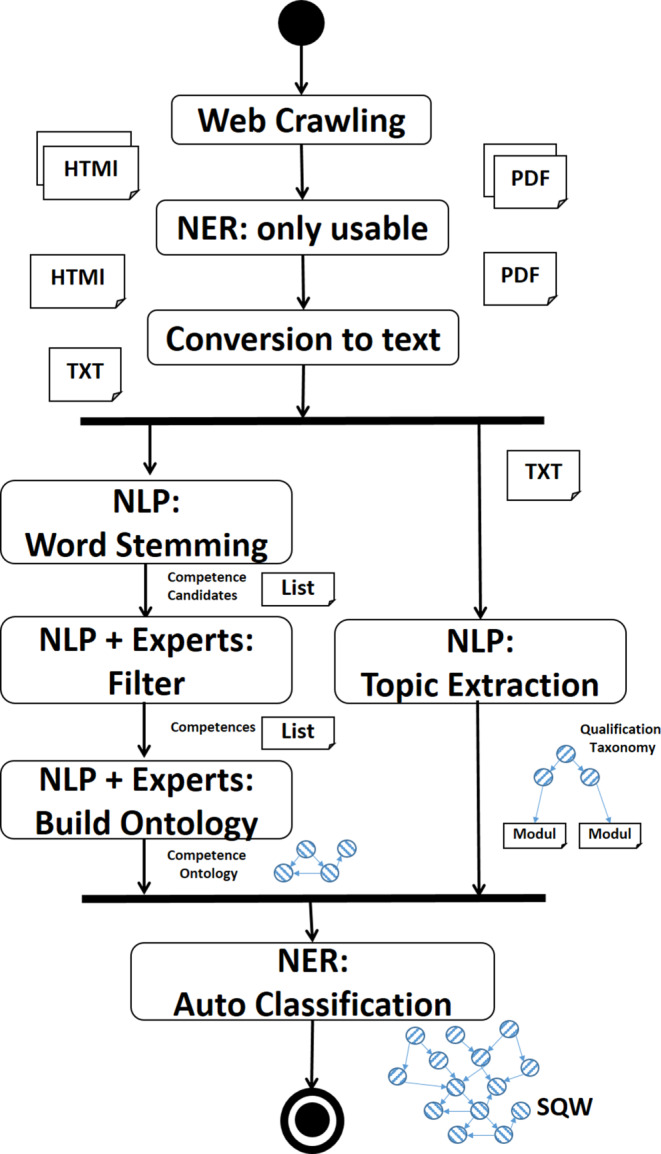
Natural language processing (*NLP*)-based process for transforming text to semantic qualification web. *HTML* HyperText Markup Language document, *TXT* Text document, *PDF* Portable Document Format document, *NLP* Natural Language Processing, *NER* Named Entity Recognition, *SQW* Semantic Qualification Web

Fig. 2 shows a simplified process for retrieving qualification offer texts from websites and transforming them into an SQW ontology. The process is shown as a UML Activity Diagram.

Web crawling can be used for systematically analyzing web pages, starting with yellow pages for qualification offers, e.g., Higher Education Compass^4^. Pages are automatically classified for relevance for the SQW, using the NLP technique of *named entity recognition (NER)* [16].

Other NLP approaches like Stemming^5^ can be used for semi-automatically extracting a competence ontology. Domain experts need to manually support by filtering and building the competence ontology. In a parallel process, the NLP approach of Topic Extraction^6^ may be used to build a qualification taxonomy.

Finally, NER-based auto-classification can be used for linking modules from the organizational taxonomy and the competencies from the competence ontology. Thus, the resulting SQW ontology connects the qualification taxonomy with the competence ontology.

This example shows how AI methods from NLP and ML can help create SeW ontologies.

### Context-aware fault diagnosis in the smart factory

The *smart factory* forms a complex environment with highly coupled, integrated, and interconnected machinery. The detection of a fault is a complex task, especially in the transition between industry 3.0 with brownfield machinery and industry 4.0. In this transition, brownfield machinery exists side by side with new *cyber-physical systems (CPSs)*. Brownfield machinery has almost no monitoring, whereas CPSs have a large amount of sensors to monitor each component and condition separately. Consequently, there may be an information overload on CPSs [17], whereas there is almost no live information available on brownfield machinery [18]. In between, there is brownfield machinery that is enhanced with internet of things (IoT) devices [19] to monitor certain conditions of the production process. As a result, there is a diverse amount and granularity of information available in current factories—some over–fulfill, some under-fulfill the information needed for the fault diagnosis process.

**Fig. 3:**
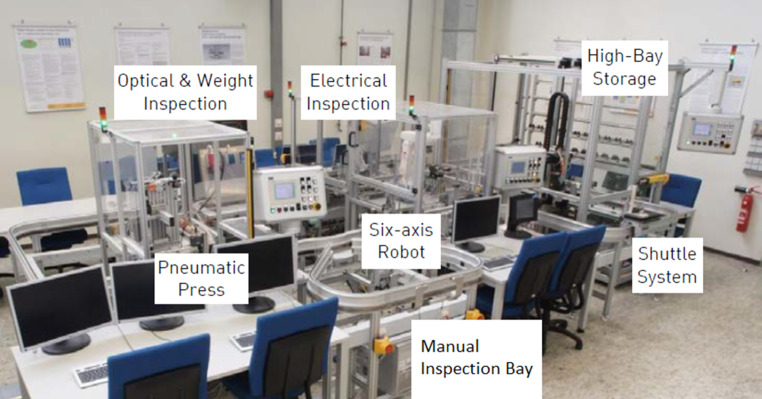
Smart factory at Darmstadt University of Applied Sciences, Germany [20]

For the fault diagnosis process, information is key to excavating the reason for a fault. The faster the problem is solved, the less costly is the production downtime. Fig. 3 shows the smart factory laboratory at Darmstadt University of Applied Sciences, Germany, where new innovations can be tested with the latest automation hardware in real-world scenarios. The smart factory produces fully functional electric relays that are used, e.g., in wind turbines. A high-bay storage with a three-axis robot lifts the unassembled relay onto a shuttle monorail system that interconnects all stations. The shuttle passes a six-axis robot that assembles the relay. Next, the pneumatic press assures connectivity between the relay and its socket. Additionally, in two inspection stations, the functionality of the relay is tested. One inspection is performed optically and by weight, the other electrically. Finally, the functioning relay will be stored back in the high-bay storage.

In such a complex environment, machine intelligence can assist the personnel in various areas to mitigate the consequences of a fault. We have published examples of automatic information extraction and information fusion to speed up the fault diagnosis process [21, 22]. Furthermore, we used autoencoder neural networks to find outliers in log data that a domain expert can use to start analyzing. In addition, we developed a semantic fusion process that is capable of semantically enriching machine events and fusing them together with documentation that guides the personnel throughout the fixing process. Guidance, or enablement of personnel, is key to solving the issues in such a complex environment. As complex the process of fault diagnosis can become, so manifold are the research directions currently in focus:

- Information provision (e.g., through the enhancement of brownfield machinery [23, 24])
- Formalization of machines, networks, and interactions, e.g., in cyber-physical systems of systems [25]
- Automatic information extraction and annotation, e.g., using machine learning to cope with big data [26]
- Assistance through intelligent visualisation, e.g., to visually pin-point faults and preconditions [27]

Addressing these research directions, we published a visual analytics (VA) model [28]. This combines production systems and their environment with computational models and visualizations to assist analysts in their daily tasks. We introduced and formalized the context as a standalone entity. We believe this will be an important entity in the fault diagnosis of the future. Consequently, we use our VA model for context-aware fault diagnosis. In addition, we published an article on a first dataset containing contextual faults to highlight the importance of the context and contextual information [20].

To conclude, analyzing the context of a fault will become more important in the future, since the complexity of smart factories will continue to increase.

### Intelligent information systems in industrial applications

An example of an intelligent information system in industrial applications supports the production process of an automotive supplier for turbochargers. A system of this kind has been implemented [29] in accordance with ISO 18828‑2 standard [30]. In this use-case, requirements such as customer-specific products and services, as well as lot-size one production, play a central role. In order to enable lot-size one production, production planning must be carried out and manufacturing needs to be transformed. Dynamically operating production lines need to react to changing demands in the shortest possible time. Outsourced production steps need to be converted to manufacturing networks with an integrated flow of information, data storage, and data access.

**Fig. 4:**
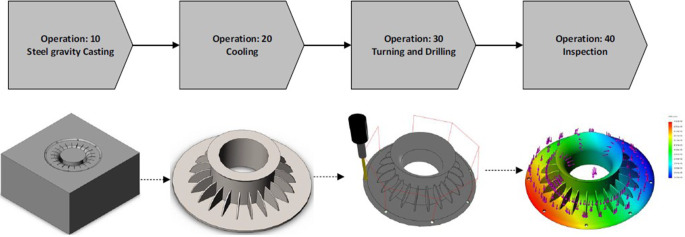
Knowledge production planning process in component manufacturing of a turbocharger housing cover

In order to meet these requirements, the *Knowledge Production Planning (KPP*^7^*) approach* ([31], see Fig. 4) with its KPP production planning ontology [32] offers the possibility to represent and store data sets in *Labeled Property Graphs (LPG*; see the section on AI technology below). KPP implementation is, among others, based on graph database Neo4j^8^ platform [33], which enables graph search with *Cypher Query Language (CQL*^9^).

Resource description framework (RDF) triple stores and LPGs both allow connected data to be explored and graphically depicted. But their models are different [34], and each have different strengths in different use cases. This has been shown in production planning use cases [35], in the fields of logistical production planning, additive production planning, and assembly production planning.

The RDF specification and model is more focused on data exchange, while LPGs are about data storage and querying. Another difference is that RDF does not offer any internal structures for nodes and edges. In contrast, LPG has these internal structures and thus enables a more detailed representation and annotation of nodes and edges. In LPGs, entities are called nodes with a unique identifier, plus a set of properties for characterization. Relationships between these nodes also have unique identifiers (IDs). Especially in LPGs, it is necessary to uniquely identify relationships to add a type and/or a set of key-value pairs in order to characterize them. “The important thing to remember here is that both the nodes and relationships have an internal structure, which differentiates this model from the RDF model” [34].

Building on this semantic formalization, and going beyond by using property graphs, KPP supports the collection, representation, administration, and reuse of knowledge related to production planning processes. In this way, KPP combines both a knowledge and a process perspective. Therefore, activities of a process with resources such as expert knowledge and documents can be commented on. These technologies offer a clearly defined formal semantic representation that supports the formal description of machine-readable production knowledge in industrial applications.

## AI methodology

*Knowledge-based AI* (a.k.a. *symbolic AI*) and *machine learning* (a.k.a. *non-symbolic AI *or *subsymbolic AI*) are the two major AI approaches that complement each other. Engineering AI applications requires sound methodological skills. In this section, we present some selected aspects.

### An ontology for machine learning

Machine learning (ML) is considered the most dominant AI approach today. Engineering professional ML applications is difficult and can be considered an art. Sound experience is mandatory and agreed-on engineering guidelines are scarce. ML libraries like scikit-learn^10^ or TensorFlow^11^, as well as integrated tools like RapidMiner^12^ or Knime^13^, offer hundreds of different ML approaches to choose from. Developers of ML applications are confronted with questions like:

- Which approaches for classification can be recommended with little training data?
- Which approaches for regression are able to deal with missing data? What are the approaches for filling missing data?
- Which prediction performance measures for classification can be recommended with unbalanced datasets?

Providers of integrated ML tools and ML libraries have started addressing the need for guidance via so-called *ML cheat sheets*. Examples include SciKit Learn—Choosing the right estimator^14^, Microsoft Azure Machine Learning Algorithm Cheat Sheet^15^, and Cheat Sheet: Machine Learning with KNIME Analytics Platform^16^. ML cheat sheets provide an overview of major ML concepts and how they interrelate. As such, they are simple ontologies, i.e., formal models of concepts and their relationships. However, they are usually restricted to one page in order to remain manageable. They may be a good guide for beginners, but cannot give a comprehensive overview of ML concepts. For detailed questions, ML engineers are obliged to search for proper documentation or follow a trial–error approach.

Wouldn’t it be handy to have an ML ontology that makes it possible to answer questions like those mentioned above? This would support ML engineers when designing ML applications. It could also be used for teaching ML. In the future, such an ontology could also be used as a knowledge base for AI applications, e.g., to support automated orchestration of ML applications (auto-modelling) or a chat bot in answering questions about ML.

First attempts at ML ontologies have been undertaken: ML-Schema^17^ provides a schema for ML ontologies to support interoperability between concrete ML ontologies. Its focus is on ML experiments, processing concrete datasets with concrete ML implementations. Overarching concepts like supervised/unsupervised/reinforcement learning are not in focus. Other attempts include OntoDM^18^, Exposé^19^, DMOP^20^, and The MEX vocabulary^21^. However, none of these fully meets the requirements for an ML ontology as outlined above.

**Fig. 5:**
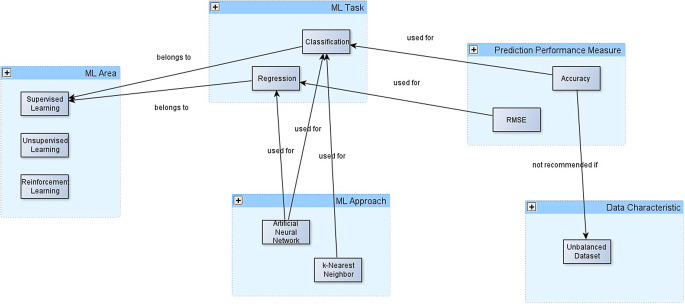
Example concepts of a machine learning (*ML*) ontology

Therefore, we formed a working group to develop such an ML ontology, potentially by interlinking existing ontologies. Fig. 5 shows some example concepts of such an ML ontology.

This example expresses that the ML approach k‑nearest neighbor can be used to classify tasks that belong to the area of supervised learning. Accuracy can be used as a prediction performance measure for classification tasks. Its use is not recommended if the data set is unbalanced. Artificial neural networks can be used for classification tasks, as well as for regression tasks—also belonging to the area of supervised learning. Root mean squared error (RMSE) can be used as a prediction performance measure for regression tasks.

The working group for building an ML ontology is, at the time of writing, in the phase of specifying goals of an ML ontology and researching related work. If you are interested in participating, please contact Bernhard Humm <bernhard.humm@h-da.de>.

### Recommendations for sound ontology modeling

Over the last 20 years, numerous recommendations on ontology modeling and meta-modeling, e.g., [36, 37], and even on bad practices [38], have been published. Criteria for sound ontologies are completeness, semantic correctness, interoperability, scalability, freedom from redundancy, compatibility to standards like RDF/OWL, and comprehensibility, e.g., by graph visualization (GV) [39].

To date, there are no truly universally accepted standards for sound ontology modeling. There is, e.g., an ongoing debate on how many levels of abstraction in a meta-model should be represented. While RDF seems to represent only one layer (everything is a resource), other approaches, including unified modeling language (UML), assume four layers (M0, … , M3), while alternatively one could differentiate between the two layers: schema layer (SL, layer of classes and meta-classes) and the layer of individuals (IL).

It is recommended that ontologies be modeled step by step: identify basic concepts (classes), data properties (intrinsic), object properties (extrinsic), define axioms. This can be performed in analogy to agile approaches to software engineering.

As in software engineering, proper naming is important in order to avoid ambiguities and increase comprehensibility of models. Guidelines for naming are available. In [40], the author proposes using different special character prefixes for the identifiers of concepts like classes, data properties, object properties, processes, and relators. This makes it possible to automatically assign different colours and shapes for the GV of ontologies. For naming of object properties, the use of nouns rather than verbs is recommended. This allows for automatic derivation of the name of an inverse property, e.g., has_employee and is_employee_of.

The use of design patterns like the materialization pattern should be enforced. Also, (RDF) reification plays an important role in modeling knowledge artifacts. Reification makes it possible to model arbitrarily nested unasserted information about situations like beliefs, wishes, intentions, etc.

The following requirements for meta-modeling formulated in [41, 42] can also be regarded as modeling recommendations. The dual facet behavior of classes should be supported, i.e., a class can be regarded as a subclass and an instance of another class at the same time. Relationships between classes and individuals should be allowed, e.g., Yo-Yo Ma is_Expert_of Violin. Dynamically adding types should be possible. Rules for the instantiation of types at different levels should be provided. Information concerning domain subjects should be described locally to avoid fragmentation and redundancy. To work with multi-level models, support for queries and navigation between levels is required.

Modeling errors should be avoided: creating cycles in hierarchies, modeling individuals as classes, or modeling classes as individuals. Reasoners like Pellet, KARMA, HermiT, etc., can help validate the syntactical correctness of ontologies.

Some members of the Dagstuhl workshop formed a working group to prepare state of art documents and a website on the topic of sound ontology modeling. If you are interested in participating, please contact Hermann Bense <hb@bense.com>.

## AI technology

In this section, we focus on one aspect of knowledge based AI, namely technologies for implementing and visualizing ontologies.

### RDF versus labeled property graphs

*Resource Description Framework (RDF)*^22^ is a W3C^23^ standard for data interchange on the web. The *Web Ontology Language (OWL)*^24^ is a language designed to represent rich and complex knowledge about things, groups of things, and relations between things. OWL is part of W3C’s semantic web technology stack, which includes RDF, RDFS^25^, SPARQL^26^, etc.

In recent years, *Labeled Property Graphs (LPG)* [34, 43] have become an important area of research and application within the semantic web community. This was mainly driven by the success of NoSQL databases like Neo4J^27^. In [43], the LPG model is defined as follows: “The labeled property graph model consists of a set of nodes V (sometimes called vertices or knowledge subjects) and edges E (sometimes called arcs or links). An edge is always related to exactly two nodes with a fixed direction from a start to an end node, defining the property graph as a directed graph. […] Both, nodes and edges, can store a set of key-value pairs, called properties and nodes can be tagged with labels additionally. Neo4J refers to edges as relationships.”

RDF and labeled property graphs both provide ways to explore and graphically depict connected data. But they are different and each has different strengths in different use cases [34].

RDF, RDFS, and OWL provide rich modeling features for the implementation of semantic web applications. On the other hand, those technologies also require a lot of “work around modeling.” RDF tuples do not allow for storing metadata properties like authors etc. [44]. Neither nodes nor edges have an internal structure. In contrast, LPG nodes and edges in Neo4J also have data properties, and nodes additionally have type information (called “labels” in Neo4J—not to be confused with labels in RDFS).

## Graph visualization

**Fig. 6:**
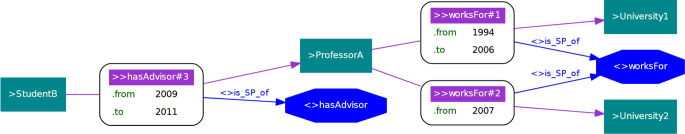
Graph visualization of relationships between students, professors, and universities

The *graph visualisation* in Fig. 6 shows an example from the education context, namely the relationships between students, professors, and universities.

The graph visualization shows that the labels of directed relationships >>hasAdvisor and >>worksFor between the individuals >StudentB, >ProfessorA, >University1, and >University2 can be annotated with any number of data properties like.from and.to. This kind of modeling follows the singleton property approach (SPA) described in [45]. It is restricted to linking precisely one subject to one object.

**Fig. 7:**
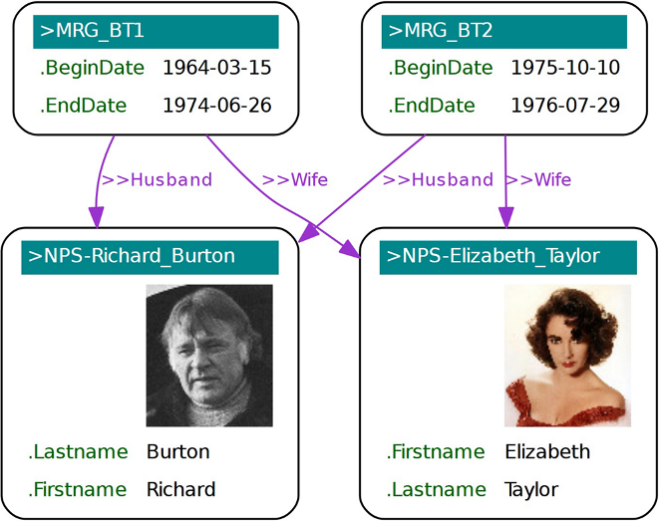
Graph visualization of marriage relationships

If another participant like the contract for the employment needs to be incorporated, then one would have to use *n*-ary relations instead. An employment subject thus could have any number of object properties like <>Employer, <>Employee and <>Contract etc. *N*-ary relations can be used to model knowledge subjects like marriage, purchase, medical treatment, etc. See, e.g., the example about marriage relationships in Fig. 7.

The relator instances >MRG_BT1 and BRG_BT2 can have any number of object properties, to associate participants like partners husband and wife. Compared to direct relationships, where the first participant is always the subject and the second is the object of the relationship, none of the participants in an *n*-ary relation is privileged. This can be equally modeled in RDF and LPG.

In cases where there are exactly two participants being related by an object property, one can choose between the representation as direct relationships or *n*-ary relations. Typical examples when direct relationships should be used are object properties indicating a direction like <>next, <>knows, <>worksFor, <>hasAdvisor etc. Typical examples of when direct relationships should not be used include when the participants in a relationship are not privileged. This is, e.g., the case with the object properties <>Husband and <>Wife in a marriage, since this would afford the redundant annotation of properties like.from and.to within both vertices.

## Conclusions

AI applications are used every day. In this article, we present some AI applications from selected application domains, namely culture, education, and industrial manufacturing. Developing AI applications requires special skills. We have presented current trends, best practices, and recommendations regarding AI methodology and technology, focusing on ontologies (knowledge-based AI) and machine learning.

The selection of approaches presented is by no means comprehensive. It reflects a subset of topics that were discussed during the 2020 online Dagstuhl workshop on applied machine intelligence.

We will continue sharing our experiences in applied machine intelligence in Dagstuhl workshops and publishing our results. If you work on intelligent applications in corporate contexts, you are cordially invited to participate in next year’s workshop. Please contact: Bernhard Humm <bernhard.humm@h-da.de> or Thomas Hoppe <thomas.hoppe@htw-berlin.de>.

## Footnotes

1 We prefer the term machine intelligence to artificial intelligence (AI) in order to avoid interpretations of AI being a form of intelligence equivalent to human intelligence. However, we will use both terms interchangeably.

2 Wikipedia: Performing arts. In Wikipedia, Accessed 6/11/2020 from https://en.wikipedia.org/w/index.php?title=Performing_arts&oldid=984658242.

3 https://www.w3.org/People/Berners-Lee/ (accessed 4 Nov 2020).

4 https://www.hochschulkompass.de/en/ (accessed 4/11/2020).

5 https://link.springer.com/referenceworkentry/10.1007%2F978-1-4899-7993-3_942-2.

6 https://towardsdatascience.com/nlp-extracting-the-main-topics-from-your-dataset-using-lda-in-minutes-21486f5aa925 (accessed 4/11/2020).

7 Knowledge Based Production Planning (KPP)—Scheme and proof-of-concept implementation, https://kpp.fernuni-hagen.de, (accessed 28/10/2020).

8 Neo4J Platform, https://neo4j.com (accessed 28/10/2020).

9 Cypher Query Language https://neo4j.com/developer/cypher-query-language (accessed 28/10/2020).

10 https://scikit-learn.org.

11 https://www.tensorflow.org.

12 https://rapidminer.com.

13 https://www.knime.com.

14 https://scikit-learn.org/stable/tutorial/machine_learning_map/index.html (accessed 20/11/2020).

15 https://docs.microsoft.com/de-de/azure/machine-learning/media/algorithm-cheat-sheet/machine-learning-algorithm-cheat-sheet.svg (accessed 20/11/2020).

16 https://www.knime.com/sites/default/files/110519_KNIME_Machine_Learning_Cheat%20Sheet.pdf (accessed 20/11/2020).

17 http://ml-schema.github.io.

18 http://www.ontodm.com.

19 https://www.openml.org/downloads/expose.owl.

20 https://raw.githubusercontent.com/agnieszkalawrynowicz/dmop.

21 http://mex.aksw.org.

22 https://www.w3.org/RDF/.

23 https://www.w3.org.

24 https://www.w3.org/2001/sw/wiki/OWL.

25 https://www.w3.org/2001/sw/wiki/RDFS.

26 https://www.w3.org/2001/sw/wiki/SPARQL.

27 https://neo4j.com.
